# Audit of nasal lysine aspirin therapy in recalcitrant aspirin exacerbated respiratory disease

**DOI:** 10.1186/1939-4551-7-18

**Published:** 2014-07-29

**Authors:** Rachel Howe, Rita M Mirakian, Prathap Pillai, Simon Gane, Yvonne C Darby, Glenis K Scadding

**Affiliations:** 1 Department of Allergy & Rhinology, Royal National Throat, Nose and Ear Hospital, ,330 Grays Inn Road, London WC1X8DA, UK

**Keywords:** Aspirin exacerbated respiratory disease, Lysine aspirin, Nasal polyposis, Late onset asthma

## Abstract

**Background:**

Aspirin – exacerbated respiratory disease can prove difficult to control. Oral aspirin desensitization is effective, but has adverse effects and may not be cardio-protective at the high doses needed.

**Objective:**

To examine the effectiveness of aspirin administered in lower doses via the nose.

**Methods:**

An audit of 121 patients with aspirin exacerbated respiratory disease (AERD), 105 of whom were treated with intranasal lysine aspirin in gradually increasing doses following positive lysine aspirin challenge.

**Results:**

Treatment was associated with subjective symptomatic improvement or stabilization in 60 of 78 patients at 3 months and 19 of 27 at 12 months. Nasal inspiratory peak flow, olfaction, exhaled and nasal nitric oxide levels were significantly improved (p < 0.05 for all). Patients with positive skin prick tests and those with later onset (>40 years) AERD improved more than non-atopics and those with early onset AERD.

Asthma outcomes over 1 year were assessed by questionnaire in 22 patients on lysine aspirin and in 20 who were positive on challenge but who either refused treatment or took it only briefly (less than or equal to 3 months). There was a significant decrease in emergency visits (p = 0.0182), hospitalization (p = 0.0074) and oral steroid use (p = 0.004) in those on nasal lysine aspirin for a year.

Gastrointestinal side effects occurred in 3.8%, lower than those reported for oral aspirin therapy. Conclusions and Clinical Relevance This form of therapy might reduce the need for expensive monoclonal antibodies in AERD patients.

## Background

Aspirin-exacerbated respiratory disease (AERD) is a difficult-to-treat chronic inflammatory disease characterised by asthma, chronic rhinosinusitis with nasal polyposis and sensitivity to aspirin and other cyclo-oxygenase 1 (COX-1) inhibiting non-steroidal anti-inflammatory drugs (NSAIDs) [1].

Patients with AERD have been shown to have high levels of pro-inflammatory molecules [2] and low levels of anti-inflammatory ones [3], leading to damage of the respiratory mucosa [4], with resulting aggressive nasal polyposis and eosinophilic asthma [5].

Ingestion of aspirin or COX-1 NSAIDs inhibit cyclo-oxygenase 1 leading to increased availability of substrate for the lipoxygenase enzymes that produce leukotrienes, further increasing pro-inflammatory mediators and reducing protective prostaglandin E2. Acute hypersensitivity reactions can occur, leading to sudden onset bronchospasm, rhinitis, laryngospasm or even death [5].

Many AERD patients are refractory to standard medical therapy, undergo numerous surgical polypectomies and require frequent oral corticosteroids for asthma [6]. Multiple open studies show oral desensitization and daily aspirin treatment can significantly improve overall symptoms and quality of life, decrease nasal polyp formation and sinusitis, reduce the need for oral corticosteroids and sinus surgery and improve nasal and asthma scores in patient with AERD at 6 months and after one year of therapy [7]. However maintenance treatment with oral aspirin should be at least 300 mg daily, ideally 325 mg twice a day [7,8], a dose associated with gastro-intestinal or other complications in 14% of patients [9]. Oral doses of aspirin over 100 mg have been recently described as not cardio-protective and possibly detrimental to the cardiovascular system [10].

Lysine acetyl-salicylate (LAS) (Synthelabo, Paris) ,the only truly soluble form of aspirin, is less likely to damage respiratory and gastric mucosae. Direct application of LAS onto involved polyp tissue means that a higher intranasal concentration can be achieved without exposing the gut or the heart to high doses. This is an open audit of the effects of topical nasal LAS on the upper and lower respiratory tract in patients with refractory AERD.

## Materials and Methods

121 patients (61 men and 60 women; mean age ± standard deviation (SD), 45.6 ± 12.6 years) with aspirin-exacerbated respiratory disease were recruited from the Rhinology Clinic at the Royal National Throat, Nose and Ear Hospital, London. All were refractory to standard medical therapy with nasal douche and intranasal corticosteroids, plus inhaled corticosteroids, β-agonists, combinations of inhaled corticosteroid plus long acting beta β-agonists and anti-leukotrienes (LTRAs). They had undergone a mean of 3.3 sinus operations. All gave written informed consent to lysine aspirin nasal challenge and verbal consent to continuation of lysine aspirin therapy at home after a positive challenge. Aspirin-sensitivity was suspected based on the patients’ histories and confirmed by nasal challenge with lysine-aspirin, as previously described [11]. This involves initial symptom scores and nasal airway measurements followed by a graduated nasal challenges with saline, followed by increasing doses of lysine aspirin starting with 5 mg aspirin equivalent ,then 10 mg, 20, 40, at 45 minute intervals, until either the patient has responded with nasal symptoms plus a 25% decrease in the nasal airway or a cumulative dose of 75 mg aspirin has been reached without any reaction. In that case oral challenge with 100 mg, then 200 mg was given. The exception was 3 patients with a convincing double positive history of previous reactions to both aspirin and another COX-1 inhibitor who did not require formal challenge [11].

Patients who consented to nasal therapy with LAS continued to take their usual medical therapy.

The project (reference 06/Q0301/6) was approved by East of England NRES Research Ethics committee.

### Dosing with lysine aspirin

Treatment was started at home on the day after the LAS nasal challenge using drops (50 ul each) from a freshly prepared 50 mg/ml solution of LAS in sodium chloride 0.9%. Written instructions for use were given to the patient together with lysine aspirin sachets, a bottle, a dropper and a 24 hour mobile number for advice. The starting dose for therapy was the dose to which the patient had responded intra-nasally on the previous day plus an extra one drop into each nostril. The patient was given instructions to increase similarly the number of drops each day, up to a maximum of nine drops in each nostril, equivalent to 45 mg of aspirin, until assessment at 3 months. The number of drops was further increased each day up to a maximum of 15–20 drops in each nostril equivalent to 75–100 mg aspirin.

Patients were warned that if they missed more than one day’s therapy they should not re- start at home, but should return to the hospital.

Exclusion criteria included pregnancy, a history of an immediate anaphylactic or urticarial reaction to aspirin or NSAID, bleeding diatheses, severe gastro-intestinal disease or patients considered unable to use such medication regularly.

The following parameters were assessed before intranasal administration: symptoms of asthma, rhinitis and nasal polyps on a visual analogue scale, nitric oxide levels in upper (nNO) and lower (e NO) airway, nasal inspiratory peak flow, smell and spirometry. Following at least 3 months of treatment, each patient was re-assessed, this was repeated at 12 months.

Sub-group analysis was performed to determine the phenotype of those who responded well to LAS treatment. Potential factors considered were:

•Anti-leukotriene response (benefit, no benefit) [12]

•Age of AERD onset (<40 y, >40 y)

•Skin prick tests (positive, negative)

#### Subjective evaluation

Each patient evaluated global treatment effectiveness based on whether their symptoms had improved, worsened or not changed. They also assessed their current symptoms of asthma, rhinitis (nasal itch, running, sneezing) and nasal polyps (nasal obstruction, sense of smell), using a validated visual analogue scale [13].

#### Objective evaluation

Recommended measures for polyp assessment including nasal airway measurement (nasal inspiratory peak flow), nasal nitric oxide and olfactory ability were used [14]. Not all patients had all measurements taken at every visit because of lack of time or staff.

### Nasal inspiratory peak flow(NIPF)

Nasal inspiratory peak flow was assessed as previously described [15], using a nasal inspiratory peak flow meter, with the best of three values being recorded.

### Nitric oxide

Nitric oxide levels were assessed by chemiluminescence using the Logan-Sinclair analyser (Logan Research, Rochester, UK). Values were taken from both sides of the nose and the lower respiratory tract, according to European guidelines [16].

### Smell

The ability to smell was scored using *Le Nez du Vin* system [17], with a maximum score of 6.

### Spirometry

Lower respiratory function was evaluated using a spirometer (Model Vitalograph 2160, Maids Moreton, UK), complying with the European Respiratory Society Recommendations [18]. The forced vital capacity, FVC (% predicted), forced expiratory volume in 1 second, FEV1 (% predicted) and FEV1/FVC(%) were recorded.

### Asthma outcomes

Asthma outcomes were evaluated by means of a questionnaire (see questionnaire at Appendix) sent to patients who had received 1 or more years of LAS therapy and to those who had a positive challenge but had not taken LAS or had received 3 months treatment or less.

### Statistical analysis

The student t-test was used to analyse the paired data using Stata 11.2. *P* values of less than 0.05 were classed as significant. Data, where applicable, are expressed as mean ± standard error of the mean.

## Results

### Patients

**Figure 1:**
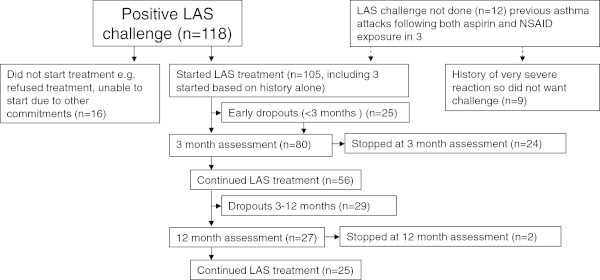
Flow diagram of patients included in audit.

Figure 1 shows a flow diagram for the patients in this audit. Sixteen patients declined LAS treatment despite a positive challenge. Of the 105 who started treatment, three had positive histories of both aspirin and NSAID sensitivity and did not require formal LAS challenge [11] so were started on 10 mg intra-nasally.

Nasal symptoms occurred on the second and subsequent doses of lysine aspirin in over 95% of patients, but were less severe than those experienced after initial challenge. In most these completely abated within days to several weeks.

Twenty five patients dropped out within 3 months, 8 within two weeks: the reasons being severe worsening of nasal symptoms- (n = 8) inability to maintain the daily regimen (n = 5) and abdominal pains (n = 2), ten patients did not return for their 3 month visit.

Treatment was stopped at the 3 month assessment in 24 patients, due to a variety of reasons including upper respiratory tract infection or acute sinusitis (n = 2), exacerbation of symptoms, particularly those of asthma (n = 2), worsening of nasal obstruction (n = 3), lack of concordance (n = 2) or lack of efficacy (n = 15).

Twenty drop outs between 3 and 12 months related to lack of efficacy or difficulty with the regime, 9 subjects were lost to follow up.

At 12 months treatment was stopped in 2 patients, one because of gastrointestinal symptoms, the other lack of efficacy.

Patients who stopped and re-started LAS treatment were excluded from the 12 month analysis, leaving 27 for assessment.

### Final doses reached

Most subjects reached 75 mg intra-nasally and continued on this dose. Two individuals were unable to increase the dose further than 9 drops in each nostril (=45 mg) because of worsening asthma at higher doses, but continued with 9 drops each side.

### GastrointestinalSide-effects

Four patients (3.8%) experienced gastrointestinal (GI) side-effects: two patients within the first couple of weeks; one each at 3 and 12 months.

### Subjective Evaluation

Table 1 shows the percentage of patients who reported improvement, worsening or no change in their symptoms at 3 months and 12 months.

**Table 1:** Subjective changes on lysine aspirin (LAS) treatment

	**At 3 months**								**At 12 months**							
	**n = 78**		**n = 78**		**n = 77**		**n = 78**		**n = 26**		**n = 26**		**n = 26**		**n = 26**	
	**Asthma**		**Rhinitis**		**Nasal polyps**		**Global**		**Asthma**		**Rhinitis**		**Nasal polyps**		**Global**	
Worse	16	21%	14	18%	24	31%	18	23%	3	12%	8	31%	6	23%	7	27%
Unchanged	38	49%	43	55%	26	34%	32	41%	10	38%	6	23%	6	23%	4	15%
Better	24	31%	21	27%	27	35%	28	36%	13	50%	12	46%	14	54%	15	58%
Better or the same	62	79%	64	82%	53	68%	60	77%	23	88%	18	69%	20	77%	19	73%

### Objective Measurements

Significant improvements were seen in NIPF at 3 months compared to pre-treatment values (145.4 ± 8.7 l/min pre-treatment and 163.3 ± 8.51 l/min at 3 months, p < 0.05), this increase was sustained at 12 months (Table 2).

**Table 2:** Objective outcomes on lysine aspirin (LAS) treatment, –Objective scores pre-treatment and at 3 and 12 months

	**Pre**	**3 m**	**12 m**
NIPF (litres/min)	145.4 ± 8.7	163.3 ± 8.5*	160.5 ± 15.9
Expired NO (ppb)	13.2 ± 1.64	14.9 ± 1.63	7.54 ± 0.85* **
Nez du Vin	1.75 ± 0.75	2.54 ± 0.80*	3.13 ± 0.64*
Nasal NO Right (ppb)	336.6 ± 32.7	347.9 ± 34.2	442.23 ± 78.7
Nasal NO Left (ppb)	343.8 ± 36.7	331.9 ± 39.3	456.4 ± 91.2

*p < 0.05 vs pre treatment.

**p < 0.05 vs 3 m.

***p < 0.05 vs 12 m.

There were improvements in nasal nitric oxide (nNO) levels in both sides of the nose at 3 and 12 months, p < 0.05 for all.

Significant changes were seen in the lower respiratory tract nitric oxide levels: at 3 months there was an increase in expired nitric oxide (eNO), but at 12 months it was significantly lower (16.3 ± 3.6 ppb pre-treatment and 7.5 ± 0.8 ppb at 12 months, p < 0.05). This reduction in eNO remains the case in patients now treated for two or more years.

There was a significant increase in the Nez du Vin smell scores at both 3 and 12 months (p < 0.05 at 3, p < 0.01 at 12 months).

Lung function measurements were not significantly affected at any time point.

### Asthma control

Data was obtained for 22 treated and 20 un-or-briefly treated patients. In the former none needed emergency or hospital asthma treatment and 4 required a course of oral prednisolone. In those who had discontinued treatment, 6 had extra primary care visits for asthma, 5 attended a hospital emergency department, 6 were hospitalized with asthma exacerbations and 13 had prednisolone courses. These figures are highly significantly in favour of lysine aspirin nasal therapy: p = 0.019, 0.007, 0.004 respectively.

### Skin prick test (SPT) positive versus skin prick tests negative patients

NIPF increased at 3 m compared to pre-treatment in those with positive SPT (143.6 ± 10.3 L/min pre- and 170.8 ± 8.1 at 3 months, p < 0.01), but not for those whose SPT were negative (152.3 ± 16.1 L/min pre- and 151.5 ± 18.7 L/min at 3 months, p = 0.48).

Smell test score improved at 3 m and 12 m compared to pre-treatment in those who had positive SPT (p < 0.01 at 3 m and p < 0.05 at 12 m), but not for those whose SPT were negative (p = 0.29 and p = 0.19 respectively).

Subjective evaluation scores for asthma in those with positive SPT tended to be better than those who had negative SPT with 83% and 94% of patients reporting asthma symptoms as better or the same at 3 and 12 months, compared to 71% and 75% at the same time-points.

### Early versus late onset AERD

Nasal inspiratory peak flow (NIPF) increased at 3 m compared to pre-treatment in those with later onset AERD (141.6 ± 10.2 L/min pre-treatment and 159.7 ± 9.9 L/min at 3 months, p < 0.05), but not for those with earlier AERD onset (157.6 ± 16.9 L/min pre-treatment and 174.7 ± 16.5 L/min at 3 months, p = 0.27).

Smell test scores were significantly higher at both 3 m and 12 months compared to pre- treatment in those with later onset AERD (p < 0.05 at 3 months and p < 0.01 at 12 months), but not for those with earlier AERD onset (p = 0.30 and 0.50 respectively).

Rhinitis symptom score 24% with later onset disease noted worsening of rhinitis at 12 months,compared to 60% with early onset.

### Anti-leukotriene (LTRA) response and outcomes

Leukotriene receptor antagonists, beneficial in some patients with asthma and nasal polyposis [6], had been previously prescribed for 96 patients, of whom 37 found benefit and continued on treatment. There was no significant difference in subjective scores and most objective data for those who found benefit from anti-leukotrienes and those who did not, with one exception : an increase in NIPF at 3 m in those for whom anti-leukotrienes were not beneficial (145.0 ± 13.8 ppb pre-treatment and 169.2 ± 12.8 ppb at 3 months, p < 0.05), not present in those on them (141.3 ± 13.5 ppb pre-treatment and 155.2 ± 13.8 ppb at 3 months, p = 0.34).

## Discussion

The patients in this audit are those with AERD refractory to standard medical and surgical therapy. AERD is a chronic inflammatory disorder of the respiratory tract [1] in which despite avoidance of aspirin and NSAIDs, mucosal inflammation of the upper and lower respiratory tracts persists and progresses [5]. Since the inflammation is progressive, a beneficial effect of treatment may be considered as lack of deterioration as well as improvement of symptoms. Based on subjective symptom evaluation the majority of patients found some benefit from nasal LAS treatment, a quarter continued regularly with a complex form of treatment for 12 months, with 73% being globally improved or stable, meaning that one patient should benefit in every 5 or 6 who are treated. This compares favourably with the number needed to treat (NNT) of 4.4 for intranasal corticosteroids, and is superior to the NNT for antihistamines which is 15.2 ,in allergic rhinitis treatment [19].

Only 3.8% of 105 patients reported gastro- intestinal side effects - approximately a quarter of the rate found with oral aspirin desensitization. There was a high drop out rate. Lack of any funding for this project meant that not all patients attended follow up visits, largely because of expense and time off work. The complexity of making up a new solution each day then putting it into the nose in the head upside down position without missing out more than a day defeated many of the remaining drop outs, None had any serious adverse event. The number of subjects known to have left because of side effects of therapy was nineteen.

However, at 3 and 12 months for all symptom groups (global, asthma, rhinitis and nasal polyps), there were some patients whose symptoms were worse than previously. Our data suggest that those with later onset disease and positive skin prick tests improve more than those with onset less than 40 years and negative skin prick tests. The reasons for this are unknown but could relate to staphylococcal enterotoxin effects which are more notable later in the disease course [20].

The effects on asthma were objectively assessed by comparing lung function tests, and exhaled nitric oxide levels before treatment and at 3 and 12 months. Exhaled nitric oxide (eNO),which reflects eosinophilic inflammation of the lower respiratory tract ,showed significant changes with an increase at 3 months that could indicate aspirin–induced mast cell degranulation in the lower respiratory tract by aspirin swallowed after nasal insertion, however this was reversed with further treatment and increased dose of lysine aspirin with a significant decrease at 12 months, maintained in patients continued on LAS therapy (data not shown). The fall in eNO at 12 months and the significantly better asthma outcomes in those on LAS therapy suggests that treatment mainly directed at the upper airway also protects the lower. However there may also be a selection bias, as those in whom the lower respiratory tract was adversely affected by LAS, and those who were non-concordant with medication, would no longer be continuing on treatment. A double blind, placebo-controlled trial would be ideal, but difficult because of blinding and funding.

The mechanism of action of LAS used in this way is uncertain and it is unlikely that patients taking 75 mg nasally are fully desensitized, though they are tolerating a dose of aspirin which is optimal for cardiovascular protection [10]. In this respect LAS is likely to be superior to the NSAID ketorolac, (the only topical form available in the USA) which is detrimental to the cardiovascular system; and to oral desensitization, where the higher doses used [7] are possibly not cardio-protective and are more likely to cause gastrointestinal bleeding. Our previous work has shown that cysteinyl LT1receptors are upregulated in AERD in nasal biopsies [21] and that the percentages of mucosal CD45 + leukocytes expressing cysteinyl leukotriene LT 1 receptors were significantly (p < 0.0001) elevated in aspirin-sensitive, but not in aspirin-tolerant patients [22]. In a small double-blind, placebo- controlled, cross over study using 16 mg LAS intra-nasally, we found a reduction in Cys LT1 receptors after 2 weeks, maintained at 6 months, compared to saline placebo [21].

In an n of 1 study with patients as their own controls using LAS at 30 mg intra-nasally in addition to routine therapy, there was significant improvement in polyp grade and NIPF [23]. Aspirin itself is an anti- inflammatory and this may be relevant, however topical aspirin was not effective in a double-blind study in aspirin tolerant(AT) polyps [24] which makes this simple explanation unlikely. It is probable that the mechanism of action relates more specifically to aspirin sensitivity and involves graduated degranulation of mast cells and eosinophils in the nasal mucosa, plus a reduction in leukotriene receptors, which we have shown previously at lower doses of intranasal lysine aspirin [21]. In addition lysine itself has activity against herpes simplex which may be implicated in AERD pathogenesis [25]. Further double-blind studies involving mediator release, mucosal genomics, biomics and proteomics are needed.

## Conclusions

This audit shows that for selected patients with refractory AERD nasal LAS treatment can reduce airway inflammation, improve symptoms, asthma outcomes and sense of smell. Advantages of nasal LAS are a reduced incidence of gastrointestinal side- effects and a dose compatible with cardio-protection when compared to oral desensitization; disadvantages include the need for daily preparation of the solution and the strict treatment regime. Since therapy with aspirin, nasally or orally is inexpensive and relatively safe it should be tried in recalcitrant AERD before monoclonal antibodies, such as anti- IgE or anti-IL5 [26,27].

## Appendix

### Topical lysine aspirin in aspirin exacerbated respiratory disease

FOLLOWUP QUESTIONNAIRE

1. Name............................................................................

2. Date of birth................................................................

PLEASE CIRCLE THE CORRECT ANSWERS BELOW

3. Gender i) Male ii) Female

4. Are you suffering from asthma? i) Yes ii) No

5. Is your asthma made worse by aspirin? i) Yes ii) No

6. Do you suffer from nasal polyps? i) Yes ii) No

7. Are your nasal polyp -related symptoms made worse by aspirin? i) Yes ii) No

8. Do you suffer from sneezing or runny nose (rhinitis)? i) Yes ii) No

9. Does aspirin worsen these symptoms? i) Yes ii) No

10. Have you undergone a lysine aspirin challenge at RNTNE Hospital? i) Yes ii) No

11. Did you start taking lysine aspirin treatment following the challenge? i) Yes ii) No

12. Have you stopped taking lysine aspirin treatment after starting it? i) Yes ii) No

13. If you have stopped; how long had you taken lysine aspirin for?

14. If you have continued how long have you been taking lysine aspirin?

15. Medications used before taking lysine aspirin:

i) Antihistamines

ii) Nasal steroids

iii) Inhaled steroids

iv) Anti-leukotrienes (montelukast)

v) Oral steroids

16. Medications used after taking lysine aspirin:

i) Antihistamines

ii) Nasal steroids

iii) Inhaled steroids

iv) Anti-leukotrienes (montelukast)

v) Oral steroids

17. How do you rate your symptoms whilst taking lysine aspirin

i) worse ii) same iii) better?

18. Have you suffered from any bad attacks of asthma in the past year? i) Yes ii) No

19. Have you had extra visits to your GP as a result of your asthma in the past year?

i) Yes ii) No

20. Have you attended A&E as a result of exacerbation of your asthma in the past year? i) Yes ii) No

21. Have you received oral steroids (Prednisolone) for exacerbation of asthma in the past year ? i) Yes ii) No

22. Have you been admitted to hospital and treated as in-patient for exacerbation of your asthma in the past year? i) Yes ii) No

## Competing interests

The author declares that they have no competing interest.

## Authors’ contributions

RH created the database of results and wrote the paper, GKS, RM and SG assessed the patients clinically, YCD organized follow up visits and made airway, nitric oxide and smell measurements, PP sent out the asthma questionnaires and analysed results, GKS oversaw the study and finalized the paper which was read and approved by all authors.

## References

[B1] Teran LM Holgate ST Park HS Sampson AP Editorial. Aspirin Exacerbated Respiratory Disease Journal of Allergy 2012 7 473863 22927868 10.1155/2012/473863 PMC3426245

[B2] Daffern P Muilenburg D Hugli TE Stevenson DD Association of urinary leukotriene E4 excretion during aspirin challenges with severity of respiratory responses J Allergy Clin Immunol 1999 7 3 559 564 10.1016/S0091-6749(99)70324-6 10482828

[B3] Sanak M Levy BD Clish CB Chiang N Gronert K Mastalerz L Serhan CN Szczeklik A Aspirin-tolerant asthmatics generate more lipoxins than aspirin-intolerant asthmatics Eur Respir J 2000 7 1 44 49 10.1034/j.1399-3003.2000.16a08.x 10933083

[B4] Stevenson DD Szczeklik A Clinical and pathologic perspectives on aspirin sensitivity and asthma J Allergy Clin Immunol 2006 7 773 786 10.1016/j.jaci.2006.07.024 17030227

[B5] Stevenson DD Sanchez-Borges M Szczeklik A Classification of allergic and pseudoallergic reactions to drugs that inhibit cyclooxygenase enzymes Ann Allergy Asthma Immunol 2001 7 3 177 180 10.1016/S1081-1206(10)62221-1 11570612

[B6] Hosemann W Surgical treatment of nasal polyposis in patients with aspirin intolerance Review Thorax 2000 7 Suppl 2 S87 S90 10.1136/thorax.55.suppl_2.S87 PMC1765953 10992570

[B7] Lee RU Stevenson DD Aspirin-Exacerbated Respiratory Disease: Evaluation and Management Allergy Asthma Immunol Res 2011 7 1 3 10 10.4168/aair.2011.3.1.3 PMC3005316 21217919

[B8] Rozsasi A Polzehl D Deutschle T Smith E Wiesmiller K Riechelmann H Keck T Long-term treatment with aspirin desensitization: a prospective clinical trial comparing 100 and 300 mg aspirin daily Allergy 2008 7 1228 1234 10.1111/j.1398-9995.2008.01658.x 18699939

[B9] Berges-Gimeno MP Simon RA Stevenson DD Long-term treatment with aspirin desensitization in asthmatic patients with aspirin-exacerbated respiratory disease J Allergy Clin Immunol 2003 7 180 186 10.1067/mai.2003.7 12532116

[B10] Steinhubl SR Bhatt DL Brennan DM Montalescot G Hankey GJ Eikelboom JW Berger PB Topol EJ CHARISMA Investigators, Aspirin to prevent cardiovascular disease: the association of aspirin dose and clopidogrel with thrombosis and bleeding Ann Intern Med 2009 7 6 379 386 10.7326/0003-4819-150-6-200903170-00006 19293071

[B11] Miller B Mirakian RM Gane S Larco J Al Sannah A Darby YC Scadding GK Nasal lysine aspirin challenge in the diagnosis of aspirin exacerbated respiratory disease Clin Exp Allergy 2013 7 874 880 10.1111/cea.12110 23889241 PMC4204273

[B12] Ragab S Parikh A Darby YC Scadding GK An open audit of montelukast, a leukotriene receptor antagonist, in nasal polyposis associated with asthma Clin Exp Allergy 2001 7 1385 1391 10.1046/j.1365-2222.2001.01160.x 11591188

[B13] Lim M Lew-Gor S Darby Y Brookes N Scadding G Lund VJ The relationship between subjective assessment instruments in chronic rhinosinusitis Rhinology 2007 7 2 144 147 17708462

[B14] Hox V Bobic S Callebaux I Jorissen M Hellings PW Nasal obstruction and smell impairment in nasal polyp disease: correlation between objective and subjective parameters Rhinology 2010 7 4 426 432 21442079 10.4193/Rhino10.049

[B15] Holmstrom M Scadding GK Lund VJ Darby Y Assessment of nasal obstruction, A comparison between rhinomanometry and nasal inspiratory peak flow Rhinology 1990 7 191 196 2251470

[B16] Kharitonov S Alving K Barnes PJ Exhaled and nasal nitric oxide measurements: recommendations, The European Respiratory Society Task Force Eur Respir J 1997 7 7 1683 1693 10.1183/09031936.97.10071683 9230267

[B17] McMahon C Scadding GK Le Nez du Vin–a quick test of olfaction Clin Otolaryngol Allied Sci 1996 7 3 278 280 10.1111/j.1365-2273.1996.tb01741.x 8818503

[B18] European Respiratory Society Standardised Lung Function Testing. Official Statement of the European Respiratory Society Eur Respir J 1993 7 Suppl 16 1 100 8499052

[B19] Portnoy JM Van Osdol T Williams PB Evidence–based strategies for treatment of allergic rhinitis Curr Allergy Asthma Rep 7 6 439 446 10.1007/s11882-004-0009-1 15462709

[B20] Bachert C Zhang N Patou J van Zele T Gevaert P Role of staphylococcal superantigens in upper airway disease Curr Opin Allergy Clin Immunol 2008 7 1 34 38 10.1097/ACI.0b013e3282f4178f 18188015

[B21] Sousa AR Parikh A Scadding G Corrigan CJ Lee TH Leukotriene-receptor expression on nasal mucosal inflammatory cells in aspirin-sensitive rhinosinusitis N Engl J Med 2002 7 19 1493 1499 10.1056/NEJMoa013508 12421891

[B22] Corrigan C Mallett K Ying S Roberts D Parikh A Scadding G Lee T Expression of the cysteinyl leukotriene receptors cysLT(1) and cysLT(2) in aspirin-sensitive and aspirin-tolerant chronic rhinosinusitis J Allergy Clin Immunol 2005 7 2 316 322 10.1016/j.jaci.2004.10.051 15696087

[B23] Ogata N Darby Y Scadding GK Intranasal lysine-aspirin administration decreases polyp volume in patients with aspirin-intolerant asthma J Laryngol Otol 2007 7 1156 1160 17697437 10.1017/S0022215107000515

[B24] Parikh AA Lysine Aspirin in Nasal Polyposis. PhD thesis 2002 University College London: Allergy & Rhinology Department

[B25] Wang X Zhang N Glorieux S Holtappels G Vaneechoutte M Krysko O Zhang L Han D Nauwynck HJ Bachert C Herpes simplex virus type 1 infection facilitates invasion of Staphylococcus aureus into the nasal mucosa and nasal polyp tissue PLoS One 2012 7 6 e39875 Epub 2012 Jun 29 10.1371/journal.pone.0039875 22768151 PMC3387208

[B26] Gevaert P Calus L Van Zele T Blomme K De Ruyck N Bauters W Hellings P Brusselle G De Bacquer D van Cauwenberge P Bachert C Omalizumab is effective in allergic and nonallergic patients with nasal polyps and asthma J Allergy Clin Immunol 2012 doi:10.1016/j.jaci.2012.07.047. [Epub ahead of print] 10.1016/j.jaci.2012.07.047 23021878

[B27] Gevaert P Van Bruaene N Cattaert T Van Steen K Van Zele T Acke F De Ruyck N Blomme K Sousa AR Marshall RP Bachert C Mepolizumab, a humanized anti-IL-5 mAb, as a treatment option for severe nasal polyposis J Allergy Clin Immunol 2011 7 5 989 995 10.1016/j.jaci.2011.07.056 21958585

